# Dielectric behaviors of Aurivillius Bi_5_Ti_3_Fe_0.5_Cr_0.5_O_15_ multiferroic polycrystals: Determining the intrinsic magnetoelectric responses by impedance spectroscopy

**DOI:** 10.1038/srep17846

**Published:** 2015-12-07

**Authors:** Wei Bai, Chao Chen, Jing Yang, Yuanyuan Zhang, Ruijuan Qi, Rong Huang, Xiaodong Tang, Chun-Gang Duan, Junhao Chu

**Affiliations:** 1 Key Lab of Polar Materials and Devices, Ministry of Education, East China Normal University, Shanghai 200241, China; 2 National Lab for Infrared Physics, Shanghai Institute of Technical Physics, Chinese Academy of Sciences, Shanghai 200083, China

## Abstract

Bismuth layer ferroelectrics (BLFs) pioneered by Aurivillius about sixty years ago have been revived recently because of the fatigue- and lead-free behaviors and high Curie temperature, and especially the robust magnetoelectric (ME) effect. However, discerning the intrinsic ME nature, and the inherence between charged defect dipole induced relaxation and spin-related behaviors are still an arduous task. Here, we report a quantitative analysis to reveal the intrinsic spin-lattice coupling in Aurivillius Cr-doped Bi_5_Ti_3_FeO_15_ (BTFCO) multiferroic polycrystals. Dielectric responses are systemically investigated by the temperature-dependent dielectric, module, impedance spectroscopy and equivalent circuit model, and two different dielectric relaxation processes occurred in grain interior of Aurivillius BTFCO polycrystals are clarified. One relaxation is proposed to associate with localized transfer of electrons between Fe^3+^ and Fe^2+^ while another one arises from the competition interaction of localized hopping of electrons between Fe^3+^ and Fe^2+^ and short-range migration of holes between Cr^3+^ and Cr^6+^. The variation of the intrinsic permittivity unambiguously confirms the coupling between spin and dipolar orderings in BTFCO polycrystals. These results offer a vital avenue for identifying the intrinsic and extrinsic signals of the electric and ME responses, and will give significant impetus to exploring the ME electronic devices of Aurivillius materials.

Bismuth layer ferroelectrics (BLFs) pioneered by Aurivillius^1^,^2^ about sixty years ago have been revived recently because of the fatigue- and lead-free behaviors and high Curie point, regarded as a potential application in ferroelectric random access memory and high-temperature piezoelectric sensors^3^,^4^. Moreover, mutliferroic (MF) properties of the coexistence of magnetic, ferroelectric and/or ferroelastic orderings in one phase can be realized by inserting the typical MF BiFeO_3_ phase into the isostructural Bi_4_Ti_3_O_12_ BLFs, whose formula is expressed as Bi_4_Bi_n-3_Ti_3_Fe_n-3_O_3n+3_ (n is an integer greater than or equal to 4, denoting the number of perovskite layer). More importantly, an associated magnetoelectric (ME) coupling between spin and dipolar orderings drives them to be qualified in the high-speed and low-power consumption multi-state memory, spintronics devices and even in photovoltaic cells^5^,^6^,^7^,^8^,^9^,^10^,^11^,^12^,^13^,^14^,^15^. Nevertheless, the nature of antiferromagnetic ordering and weak ME coupling of Aurivillius MF phases restricts their prospects of the practical application^6^,^7^,^8^,^9^,^10^,^11^,^12^,^13^,^14^. Consequently, various routes including A/B site doping, interface and stress engineering were adopted to regulate the magnetic orderings and ME coupling^5^,^6^,^7^,^8^,^9^,^10^,^11^,^12^,^13^,^14^. For instance, room-temperature ferromagnetic along with ferroelectric properties was reported for materials based on the Bi_5_Ti_3_FeO_15_ (BTFO) with half of Fe cations substituted by either Co or Ni ions^8^,^9^. Rich magnetic and electrical behaviors were observed in Bi_6_Ti_3_Fe_2_O_18_ by various doping processes^10^,^11^,^12^. In particular, magnetic-field-induced ferroelectric switching was achieved in the Bi_6_Ti_2.8_Fe_1.52_Mn_0.68_O_18_ film^13^. And a robust room-temperature ME coupling of ~400 mV/Oe.cm was yielded in the magnetically short-range ordered BTFO films^5^, and an obvious magnetodielectric (MD) effect (~10.5% at room temperature) was further optimized in La and Co co-doped BTFO phase^6^. These would greatly promote the ME/MD device applications based on the Aurivillius MF materials.

However, directly stating the coupling between spin and lattice by measuring the effect of the electric field on the magnetization or the effect of the magnetic field on the polarization is an arduous task because many MF materials are poor insulators, preventing a sufficient electric field being applied^16^,^17^,^18^, and hampering the determination of ME coupling in experiment. An alternative route has therefore been chosen to explore the ME character, consisting of studying the change of the permittivity *ε* either by applying a magnetic field, *i.e.* the so-called magneto-capacitance effect or MD effect, or searching for the anomaly of *ε* in the temperature dependence of *ε*(*T*) close to the magnetic transition point^19^,^20^,^21^,^22^,^23^,^24^,^25^. Moreover, confirming an intrinsic *ε* of MF materials is also a great challenge by itself because of the fact that the charged defect dipole induced by a leakage nature, and parasite capacitances formed at the interface between film and electrodes or grain boundaries contribute to the dielectric responses. As it has been proposed, in particular, the associated interface-driven MD effect due to the structural inhomogeneity will significantly obscure the intrinsic coupling between magnetic and electrical polarization^22^. Note that complex impedance spectroscopy is a powerful technique to analyze the microstructure-property relationship^14^,^25^, which is beneficial to distinguish an intrinsic (bulk) signal from an extrinsic one (grain boundary, and/or electrode contact). Therefore, a quantitative analysis by the magneto-impedance spectroscopy is indispensable in Aurivillius MF phases, which could shed light on the physical mechanism of the ME/MD coupling elusive in Aurivillius MF materials^5^,^6^,^9^,^13^.

In addition, there inevitably appear Bi volatilization, carrier transfer/hopping in the transitional metals and oxygen vacancies. Especially, the multi-valence configuration of Ni, Mn, Fe, Cr and Co cations would bring about creation of plausible electrical heterogeneities^14^,^26^,^27^,^28^,^29^,^30^. Accordingly, polarizability along with conductivity simultaneously occur when these multi-valence cations are included in oxides, where electron/hole migration between the mixed valence states is usually involved, which plays a predominant role in determining the electrical properties. It is thus crucial to clarify how the structural defects correlate with the electrical properties. Moreover, discerning the effects of the defects avails providing insights into the migration kinetics of charged defects and deep understanding the inherence between the defect carriers and dielectric and ME/MD responses.

In this work, systematic studies are conducted on the temperature and magnetic field dependent dielectric/impedance spectroscopy to explore the dielectric and MD responses in Aurivillius Cr-doped BTFO (BTFCO) polycrystals. On one hand, the physical nature of the present relaxation processes in association with the transfer/hopping defect carriers due to the multivalent configuration of B-cations is addressed, which benefits to reveal the inherence between charged defects and dielectric responses. On the other hand, the permittivity and resistance are quantitatively extracted by analyzing the electrical responses of grains and grain boundaries, which makes a interpretation of the microscopic process discerning an extrinsic and extrinsic MD effects, yields a quantitative correlation between the dynamic processes of charged defect induced relaxation and magnetic related behaviors, and further avails clarifying the physical mechanism of spin-lattice coupling in Aurivillius MF materials.

## Results and Discussion

**Figure 1:**
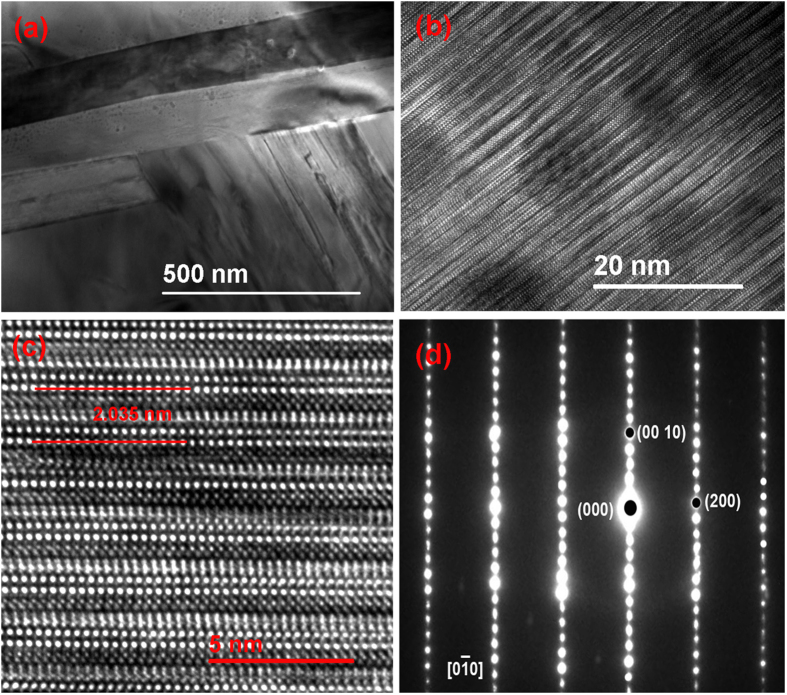
(**a–c**) show the bright-field HRTEM images at different magnifications, and (**d**) the SAED pattern of the BTFCO polycrystals.

Figure 1(a–c) show the high-resolution transmission electron microscopy (HRTEM) images of the BTFCO polycrystals at different magnifications. Layer-like grains with random orientations are closely stacked as given in Fig. 1(a), and the typical Bi-layered structure is observed as indicated in Fig. 1(b). The four-layer Aurivillius structure is further identified by the well-defined electron diffraction pattern (EDP), where three Ti-O layers and one Fe(Cr)-O layer are sandwiched by two Bi_2_O_2_ layer with a spacing of ~2.035 nm as shown in Fig. 1(c). The lattice constant *c* is thus calculated to be 4.07 nm, in line with the X-ray diffraction data^31^,^32^. Additionally, EDP also indicates the intact crystal texture of Aurivillius BTFO phase after the incorporation of Cr ions, whereas the distribution information of the Fe and Cr ions is unavailable. The selected area electron diffraction (SAED) patterns of the [0
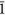
0] zone in Fig. 1(d) reveals a single crystal nature at least in the selected area supporting the excellent crystallization of the BTFCO polycrystals.

**Figure 2:**
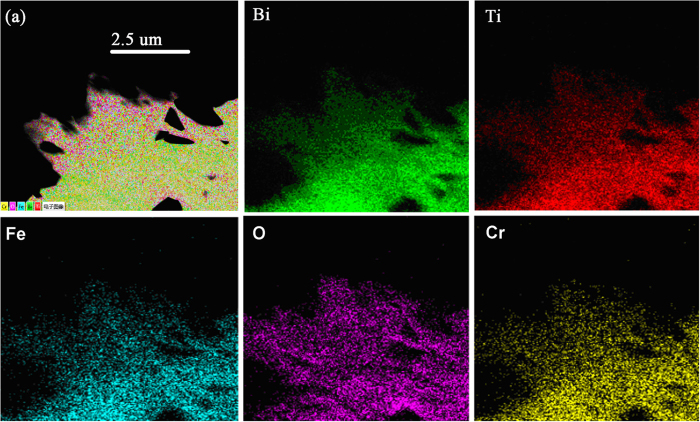
TEM images and the corresponding EDX mapping of the BTFCO polycrystals.

The composition analysis of the BTFCO polycrystals is further determined by the energy dispersive X-ray (EDX) technique. Figure 2(a) shows the dark-field TEM image of a randomly selected area. Uniform chemical composition mappings of the respective Bi, Ti, Fe, O and Cr elements given in Fig. 2 confirm the homogeneity of these element distributions, further supporting the incorporation of Cr ions into B (Fe/Ti) sites and the obvious absence of impurity in the BTFCO polycrystals.

**Figure 3:**
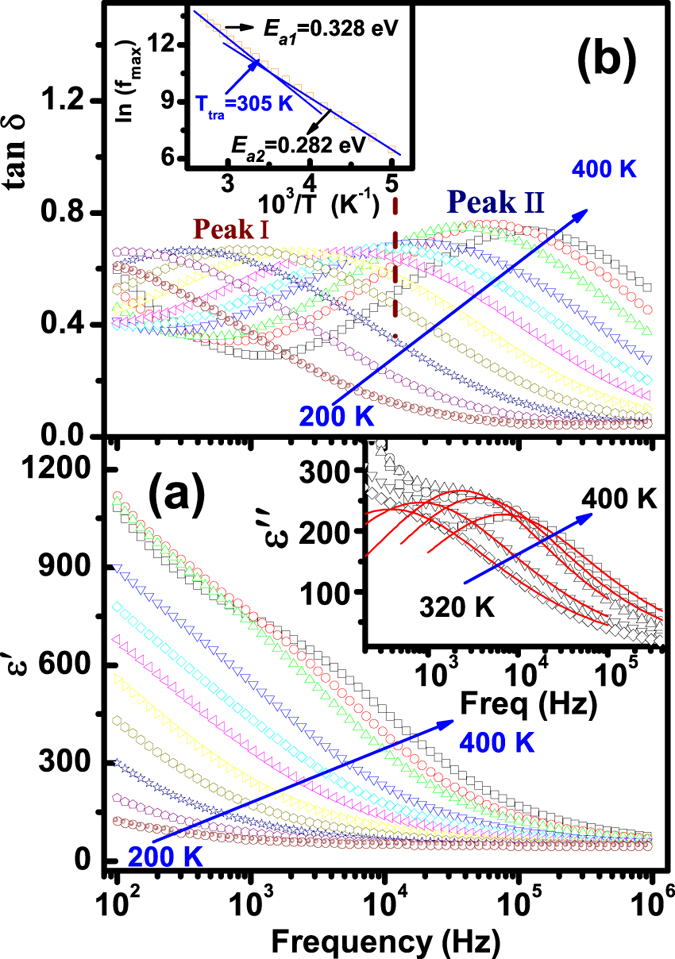
The frequency dependency of (**a**) *ε*′ and (**b**) *tanδ* for the BTFCO polycrystals at various temperatures. The solid lines are fitted curves to the Eq. (1) in the inset of Fig. 3(a), and inset of Fig. 3(b) shows the temperature dependence of relaxation time.

Figure 3 shows the permittivity (*ε*′) and loss tangent (*tanδ*) as a function of frequency at various temperatures from 200 to 400 K of Aurivillius BTFCO polycrystals. A plateau independent of temperature is found at high frequency as illustrated in Fig. 3(a), indicating an intrinsic dielectric response. However, notable increase in *ε*′ with decreasing frequency and increasing temperature in Fig. 3(a) indicates a strong dielectric dispersion and a large contribution of thermally-activated charges, such as space charges, charged defects and related defect complex. And long-range and/or localized migration of these defect carriers is usually accompanied by dielectric relaxation process as confirmed by the presence of loss peaks in Fig. 3(b). Moreover, one can see that the plateaus appeared in *tanδ* curves have two different peak magnitudes below and above ~10^4^ Hz as indicated by Peak I and Peak II in Fig. 3(b). These peaks have a shift to a higher frequency with increasing temperature, behaving as a typical characteristic of dielectric relaxation phenomenon. Moreover, Ln(*f*_*relax*_) *vs.*10^3^/*T* [here *f*_*relax*_ denotes the peak *tanδ*] is plotted in inset of Fig. 3(b) to explore the relaxation mechanism by the Arrhenius relation, *f*_*relax*_ = *f*_*0*_exp(*E*_*relax*_/*k*_*B*_*T*). Here *f*_*0*_ is a prefactor, *E*_*relax*_ represents the relaxation activation energy calculated from complex modulus, *k*_*B*_ is the Boltzmann constant and *T* is the absolute temperature. To further evaluate the physical nature of the relaxation process with definite peaks, the dielectric data are quantitatively analyzed by the Cole-Cole equation^33^,


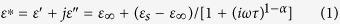


where *ε*_*s*_ and *ε*_*∞*_ are respectively the static and high-frequency permittivity, *ω* is the angular frequency, *τ* denotes the mean relaxation time and *α* is the angle of the semicircular arc. The dielectric loss peaks can well be described by Eq. (1) as plotted in the inset of Fig. 3(b). The values of *α* are therefore calculated to be 0.65–0.76 (1 for the ideal Debye model), implying a Debye-like relaxation behavior. The dynamics of the relaxation process can be described by the mean relaxation time *τ* by the Arrhenius relationship, *τ* = *τ*_*0*_ exp(*E*_*a*_/*k*_*B*_*T*). Here *τ*_0_ is the prefactor, *E*_*a*_ is the relaxation activation energy, *k*_*B*_ is the Boltzmann constant and *T* is the absolute temperature. Elaborately, note that two slopes along with *E*_*a2*_ = 0.282 eV at low temperature (T < 305 K) and *E*_*a1*_ = 0.328 eV at high temperature (T > 305 K) rather than one can better describe the plot of *Ln*(*τ*) *vs.* 10^3^/*T* as shown in inset of Fig. 3(b), implying that two relaxation behaviors probably appear in the BTFCO polycrystals.

**Figure 4:**
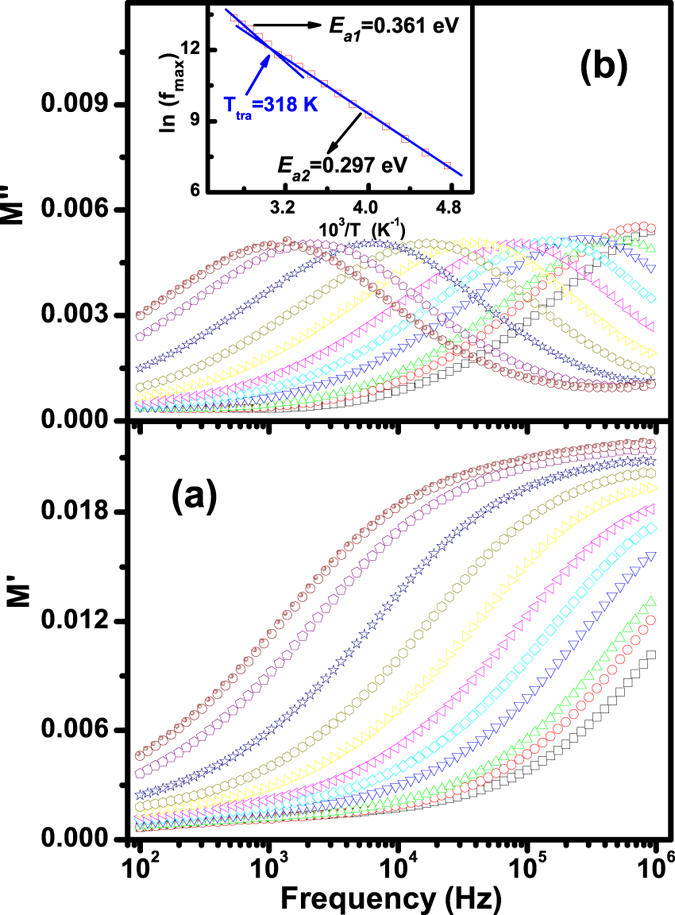
Frequency dependence of *M*′ (**a**) and *M*″ (**b**) at temperatures from 200 to 400 K. Inset in Fig. 4(b) is the plot of relaxation frequency versus 10^3^/*T*.

Note that electric module (M^*^) scales inversely to the complex permittivity^14^,^27^, that is the more conductivity loss contributes to the permittivity, the less conductivity loss affects the module. The frequency dependency of the real *M*′ and imaginary *M*″ parts of M^*^, M^*^ = *M*′ + *jM*″ = 1/ε^*^ = (*ε*′ + *jε*″)/|*ε*|^2^, are plotted in Fig. 4. One can see from Fig. 4(a) that two plateaus appear in the *M*′–*f* curve at low temperatures and the values of *M*′ increase with frequency. And a well-defined *M*″ peak appears as expected corresponding to a typical relaxation nature, implying a shift toward a lower frequency upon decreasing temperature as shown in Fig. 4(b). Here the relaxation frequency (*f*_*relax*_) corresponding to *M*″ peak indicates the transition from long-range to short-range migration with increasing frequency. *Ln*(*f*_*relax*_) vs 10^3^/*T* is also plotted [inset in Fig. 4(b)] to explore possible relaxation mechanism by the Arrhenius law, *f*_*relax*_ = *f*_*0*_ exp(−*E*_*relax*_/*k*_*B*_*T*), where *f*_*0*_ is the pre-exponential term, and *E*_*relax*_ is the relaxation activation energy. The plot can also be divided into two sections with *E*_*a1*_ = 0.297 eV below ~314 K and *E*_*a2*_ = 0.361 eV above ~314 K, in line with those of the frequency dependence of permittivity. These data further indicate that two probable relaxation processes indeed occur in the BTFCO polycrystals.

**Figure 5:**
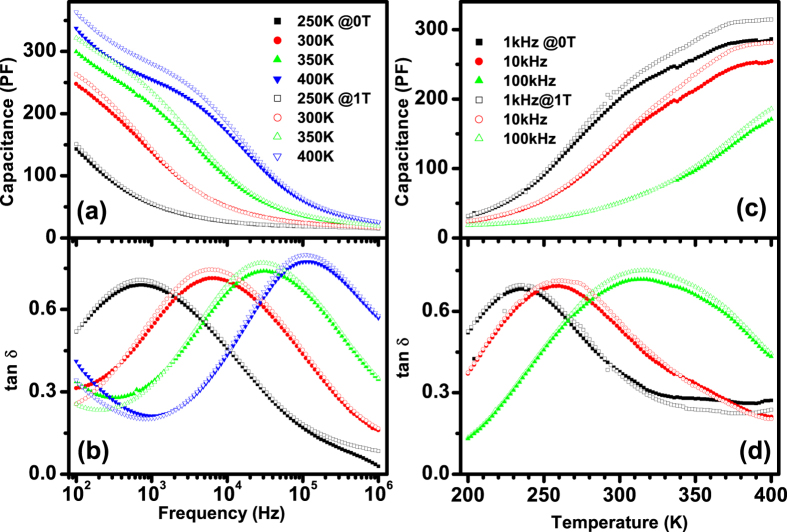
The frequency dependency of *ε* (**a**) and *tanδ* (**b**) at different temperatures, and *ε* (**a**) and *tanδ* (**b**) as a function of temperature at various frequencies of the BTFCO polycrystals at 0 and 1 T.

The frequency dependency of the *ε* and *tanδ* at different temperatures, and the *ε* and *tanδ* as a function of temperature at various frequencies of the BTFCO polycrystals at 0 and 1T are respectively shown in Fig. 5(a–d). Some points are addressed about the effect of the applied magnetic field on the dielectric behaviors, henceforth denoted as magneto-dielectric (MD) effect. 1) The enhancement of both the dielectric constant and loss with applied magnetic fields implies that the dominant mechanism of the MD effect might be an intrinsic effect rather than the Maxwell-Wagner effect. Note that the variation of the dielectric constant and loss is opposite with applied magnetic field assumed that the MD coupling results from the MW effect and magnetoresistance 2)^22^,^24^. The MD coupling at low frequencies are more apparent than those in high frequency region as indicated in Fig. 5(a,c), implying the existence of a possible extrinsic parasitic contribution^22^,^24^, such as interfacial effect between grain boundaries and/or sample and electrodes. However, the MD effect weakens with increasing frequency while still observed at 100 kHz in Fig. 5(c), revealing an intrinsic spin-lattice coupling. These data also exhibit the competition between the intrinsic and extrinsic MD effects. 3) The MD coupling enhances as the temperature rises as shown in Fig. 5(a,c). Interestingly, 4) the MD effect almost coincides with the onset of the dielectric relaxation in Fig. 5(b), and remarkable variation of dielectric loss with the magnetic field occurs close to the loss peaks as displayed in Fig. 5(c,d), implying a correlation of defect dipole induced relaxation and spin related behaviors. Nowadays, a question arises: what are the mechanisms of the present dielectric relaxation process and MD coupling?

**Figure 6:**
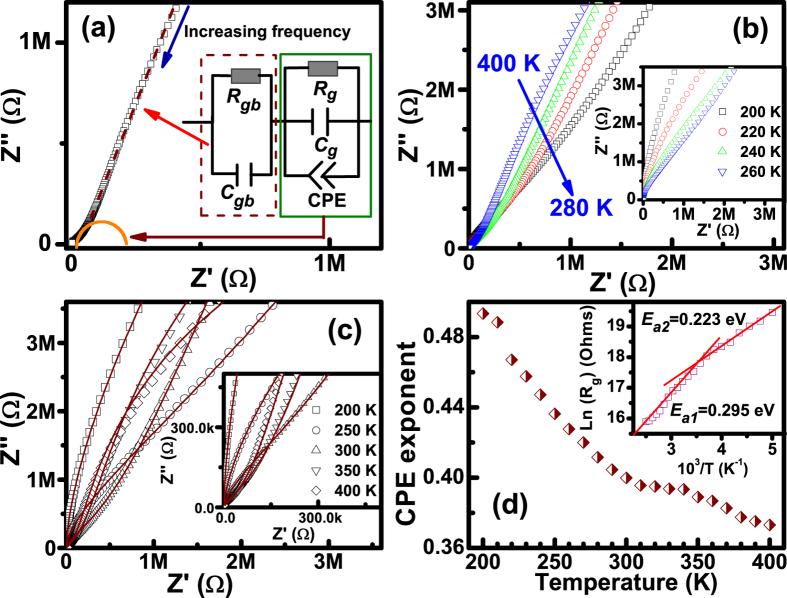
(**a**) Impedance complex plane (−*Z*″ − *Z*′ plots) data at 380 K. The visual guide of the dashed line indicates the two semicircular at high and low frequency. The inset show the equivalent circuit model, describing the different electrical responses occurred in BTFCO polycrystals. (**b**) and the inset show the −*Z*″ − *Z*′ plots at various temperatures. Experimental data (open symbols) and fitting (solid lines) using the proposed equivalent circuit model of some illustrative temperatures. The inset zooms in on the high-frequency region. (**d**) Temperature dependent CPE exponent, and the inset is the plot of the fitting Ln(*R*_*g*_) versus 10^3^/*T*.

The complex impedance data obtained at different temperatures from 200 to 400 K are shown as Cole-Cole diagrams in Fig. 6. Two poorly resolved semicircular arcs at 380 K can clearly be distinguished as illustrated in Fig. 6(a), which are usually ascribed to the contributions of grains (G) and grain boundaries (GB), respectively^14^,^20^,^30^. Clearly, the radius of the low-frequency semicircle is much larger than that of the high-frequency one, reflecting the large difference in magnitudes of resistance between G and GB. Inspection of the impedance data at various temperatures shown in Fig. 6(b) and inset of Fig. 6(b) reveals that the high-frequency semicircular arc significantly decreases with increasing temperature, evidencing that the resistivity of the high-frequency response decreases as the temperature rises.

To further establish a connection between microstructure and electrical properties and obtain reliable values of the electrical parameters, an equivalent circuit model based on brick-layer model is established shown in the inset in Fig. 6(a)^34^. Here, *C*_*gb*_is the capacitance related to the GB layer, *R*_*gb*_ represents the resistance across the GB layer, *C*_*b*_denotes the capacitance related to domain and dipole reorientation in grain, *R*_*g*_is the resistance associated with the grain, and CPE is a constant phase element implying the nonideal dielectric response correlating the *ac* conductivity with the movement of charge carriers in the grains^14^,^34^, implying the departure from ideal Debye behavior. The CPE admittance is *Y*_*CPE*_ = *Y*_*0*_ (*jω*)^n^ = *Aω*^*n*^ + *jBω*^*n*^ along with,


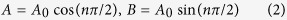


where *A*_*0*_ and *n* are only the temperature-dependent parameters, *A*_*0*_ confines the magnitude of the dispersion and 0≤*n*≤1. The parameter *n* is equal 1 for ideal capacitor and equal 0 for ideal resistor^35^. Our data are then simulated by ZVIEW version 2 employing the above-proposed equivalent circuit model. A close agreement between the experimental data (symbols lines) and model (solid lines) is indicated by the Cole-Cole diagrams in Fig. 6(c), corroborated by the low goodness-of-fit indicator *χ*^*2*^ which is ~10^−3^ in all temperature ranges. The fitting results indeed exhibit that *R*_*gb*_is far higher than *R*_*g*_. The CPE exponent *n*, which decreases upon increasing temperature [Fig. 6(d)], pointing to an enhanced nonideality of the dielectric behavior on heating, indicates a defined anomaly of ~316 K, closely coinciding with the transition point from the Arrhenius dependences of *τ* and *f*_*relax*_ in formalisms of *tan δ* and *M*″. The inset of Fig. 6(d) shows the plot of *Ln*(*R*_*g*_) versus 10^3^/*T*, which is also divided into two sections with *E*_*a1*_ = 0.226 eV and *E*_*a2*_ = 0.304 eV, further suggesting that both the two relaxation processes occur in grain interior instead of the responses of G and GB in the BTFCO polycrystals. As a consequence, charged defect associated with dipole reorientation and localized/long-range migration in grain interiors account for the involved relaxation behaviors.

The fitting *E*_*a2*_for the high temperature region is found to be close to the previously reported activation energy values of ~0.29 eV in LuFe_2_O_4_^16^, ~0.38 eV in Sr(Fe_0.5_Nd_0.5_)O_3_^17^, ~0.3 eV in BiFeO_3_^26^,^27^ and ~0.326 eV in Bi_6_Ti_3_Fe_2_O_18_^14^, attributed to the electron transfer/hopping between Fe^2+^ and Fe^3+^. Whereas note that the fitting *E*_*a1*_ is quite consistent with the *E*_*a*_ of the conductivity of 0.25 eV in LaCrO_3_, 0.23 eV in PrCrO_3_, 0.31 eV in NdCrO_3_, 0.38 eV in SmCrO_3_, 0.32 eV in EuCrO_3_, and 0.25 eV in GdCrO_3_ polycrystals^28^, ascribed to the hopping of holes between Cr^3+^ and Cr^4+^, i.e., X^3+^ → X_0_ = 3[h] and Cr^3+^+[h] → Cr^4+^. However, the presence of Cr^6+^ ions has been reported in Gd-doped LaCrO_3_ orthochromite^29^, where the values of *E*_*a*_ range from 0.21 eV to 0.39 eV with Gd-doping content from 0 to 0.2, derived from the hopping of holes between Cr^3+^ and Cr^6+^: Cr^3^+3[h] → Cr^6+^. Accordingly, the similar activation energies from the electron and/or hole hopping of Fe or Cr ions with multi-valence states would take a great challenge to distinguish the different mechanisms. For example, only single activation energy of the Cr-doped GaFeO_3_ compounds has been found with the same values ∼0.22 eV and 0.27 eV for Cr = 0.1 and 0.15, where the relaxation process is attributed to the same type of charge carrier^30^. Hence, these data indicate that one relaxation process at higher temperatures is likely caused by the dielectric response of equivalent bipolar reversal and lag effects associated with the electron hopping between Fe ions with various valence states while another one in low temperature region might root from the hole transfer of Cr ions because of the multi-valence states.

**Figure 7:**
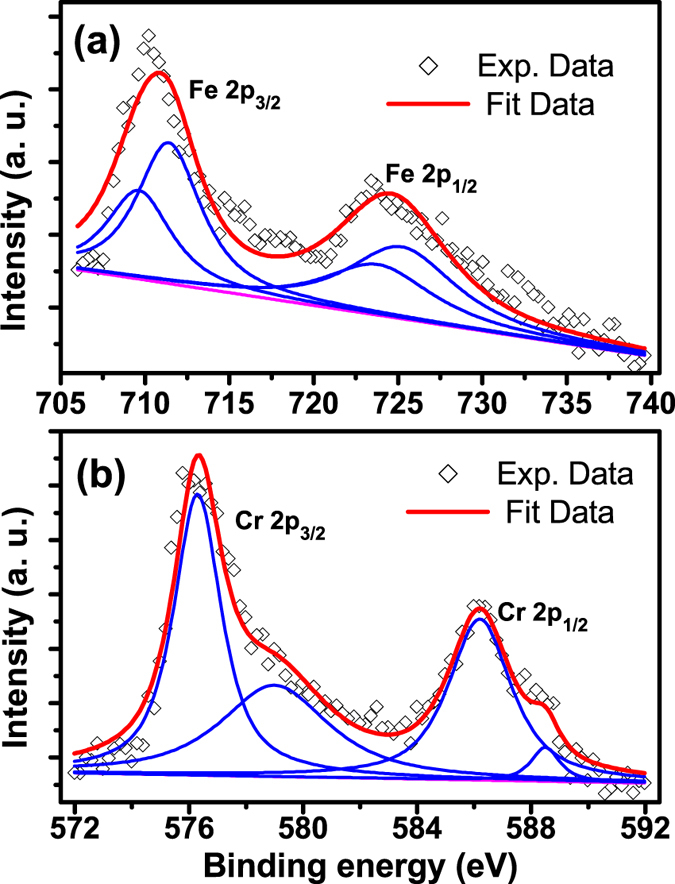
XPS spectra for Fe 2p (**a**) and Cr 2p (**b**) signals (open symbols) of the BTFCO polycrystals. Solid lines are the fitting data deconvoluted after background subtraction using XPS standard software.

To verify the optimal oxide states in the BTFCO polycrystals, we investigate the XPS spectra for Fe 2p and Cr 2p signals of the BTFCO polycrystals. The XPS spectra for these signals can be deconvoluted after background subtraction by fitting using XPS standard software. Figure 7(a) shows the Fe 2p core-level spectra of the BTFCO polycrystals, which can be well described by the Fe^3+^ and Fe^2+^ ions. The binding energies at 711.3 eV and 724.8 eV are respectively referenced by Fe_2_O_3_^35^ and FeOOH^36^ while the ones located at 709.5 eV and 723.4 eV are comparable to those in FeO^35^, indicating that the Fe ions in the BTFCO polycrystals are the coexistence of 3+ and 2+ valence states. Furthermore, Fig. 7(b) displays the Cr 2p3/2 and Cr 2p1/2 XPS spectra with the respective binding energies 576. 3 eV and 586. 2 eV^36^,^37^, and 579. 0 eV and 588. 5 eV^38^, which are well matched by Cr^3+^ and Cr^6+^ oxidation states after a least square process referenced by Cr_2_O_3_ and CrO_3_ phases, respectively, illustrating that the contribution of the Cr ions in the BTFCO polycrystals arises from Cr^3+^ and Cr^6+^ ions. Therefore, these results confirm that that one relaxation process at higher temperatures can be attributed to the electron hopping of Fe ions between Fe^+3^ and Fe^2+^ ions while another one in the low temperature region is dominated by the hole transfer of Cr ions between Cr^+3^ and Cr^6+^ ions occurred in the grain interiors.

**Figure 8:**
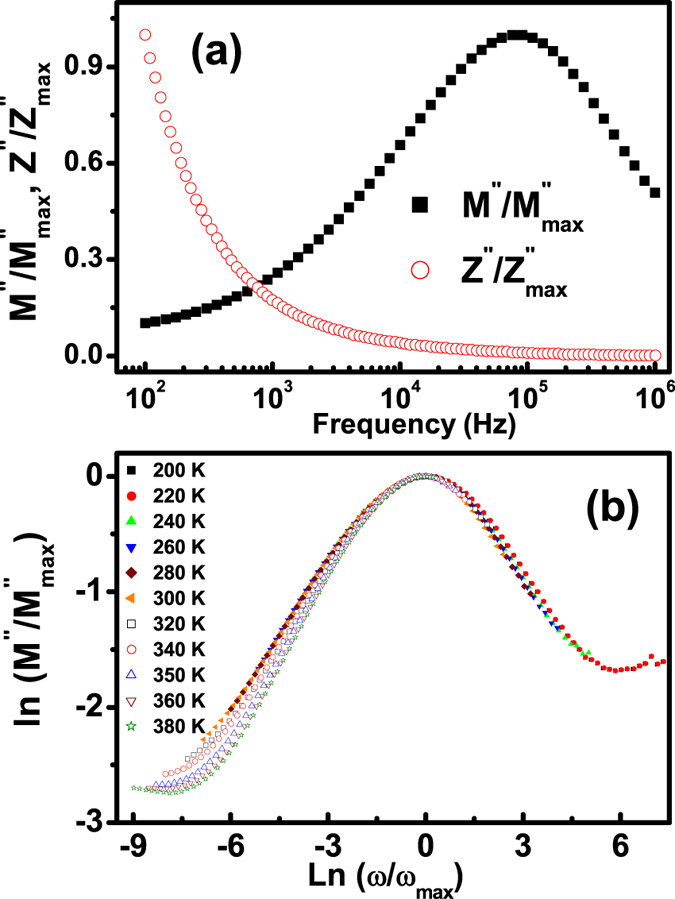
(**a**) Normalized *M*″/*M*″_*max*_ and *Z*″/*Z*″_*max*_ as a function of frequency at 320 K. (**b**) Scaling behaviors at various temperatures for electron hopping of Fe ions between Fe^+3^ and Fe^2+^ ions and the hole transfer of Cr ions between Cr^+3^ and Cr^6+^ ions.

The normalized functions of *M*″/*M*″_*max*_ and *Z*″/*Z*″_*max*_ at the typical temperature of 320 K are given in Fig. 8(a) to distinguish that the relaxation processes are dominated by the short-range or long-range movement of charge carriers^14^,^27^. The remarkable separation between the normalized *M*″ and *Z*″ peak implies the current relaxations are dominated by the localized hopping of charge carriers^14^,^27^, and departed from ideal Debye model consistent with that derived from Eq. (1) and the proposed equivalent circuit model plotted in the inset of Fig. 6(a). These results show that the electron hopping of Fe ions between Fe^+3^ and Fe^2+^ ions, and the hole transfer of Cr ions between Cr^+3^ and Cr^6+^ ions are regarded as the localized hopping of charge carriers in the grain interiors. Moreover, the plots of *M*″/*M*″_*max*_ versus log (*ω*/*ω*_*max*_) at different temperatures, where *ω*_*max*_ denotes the peak frequency, are shown in Fig. 8(b) to further study the scaling behaviors. The data below ~300 K are completely collapsed into one curve, indicating that the dynamic process of the hole transfer of Cr ions between Cr^+3^ and Cr^6+^ ions occurring at various time scales shows the same activation energy and the distribution of relaxation times is temperature independent^14^,^27^. In contrast, the entirely different spectra above ~300 K in Fig. 8(b) reveal that the distribution of relaxation times related to the carrier hopping possibly depends on the temperature, which is indicative of competition of the localized hopping of electrons between Fe^+3^ and Fe^2+^ ions, and holes between Cr^+3^ and Cr^6+^ ions at higher temperature region. The temperature transition point at about 300 K as shown in Fig. 8(b), similar to those of the anomaly of the CPE exponent *n* [Fig. 6(d)], and the Arrhenius dependences of *f*_*max*_ in formalisms of *tanδ* [inset in Fig. 3(a)], *M*″ [inset in Fig. 4(a)] and *R*_*g*_ [inset in Fig. 6(d)] from the equivalent circuit model, further confirms that one of the two relaxations at low temperatures is dominated by the localized transfer of the hole between Cr^+3^ and Cr^6+^ ions while another one in high temperature region is dependent on the completion of localized hopping of the electron between Fe^+3^ and Fe^2+^ ions and short-range migration of the hole between Cr^+3^ and Cr^6+^ ions.

**Figure 9:**
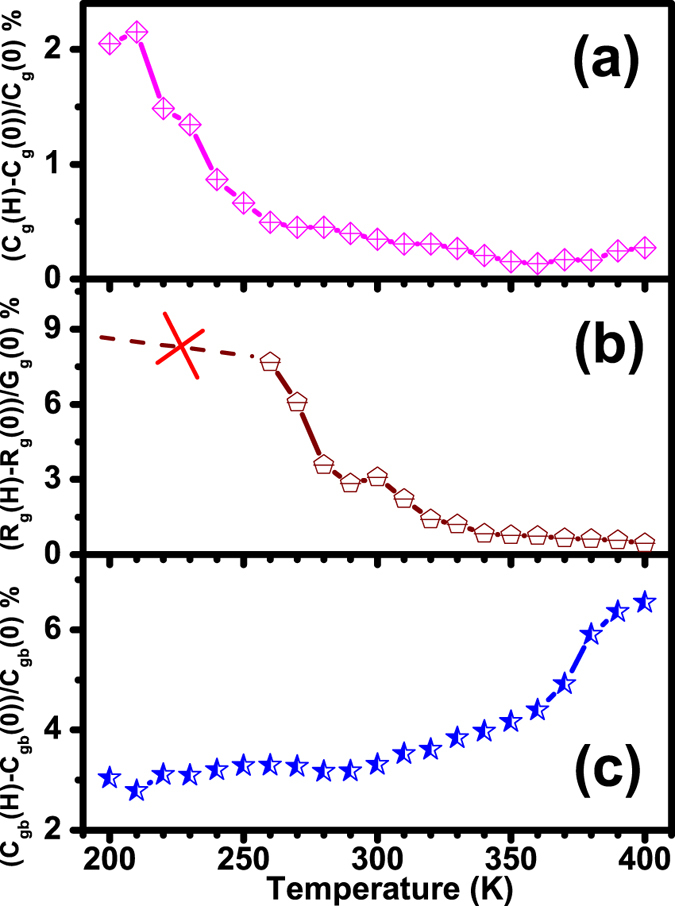
The variation, defined as [*M*(*H*)-*M*(*0*)]/*M*(*0*) × 100%, *M* denotes *R*_*g*_, *C*_*g*_ and *C*_*gb*_, of *C*_*g*_(**a**), *R*_*g*_(**b**) and *C*_*gb*_(**c**) values at 0 and 1 T obtained by magnetoimpedance spectroscopy.

The enhancement of both the dielectric constant and loss with applied magnetic fields implies that the dominant mechanism of the MD effect is highly likely an intrinsic effect, based on the fact that the variation of the dielectric constant and loss is opposite with the magnetic field assuming that the MD behavior results from the Maxwell-Wagner effect and magnetoresistance^22^,^24^. Generally, the origin of MD effect is closely correlated with the magnetic transition point in single-phase multiferroics. Besides, the MD effect is found close to the dielectric peak in BTFO^39^, La-doped BiMnO_3_^19^ and La_2_NiMnO_6_^20^ materials, but the mechanism of MD coupling is a little ambiguous. To clarify the possible origin of the observed MD coupling, the equivalent circuit model proposed in the inset in Fig. 6(a) is also used to fit the impedance spectra at 1T to evaluate the respective contributions of resistivity and capacitance from the grain and grain boundaries of the BTFCO polycrystals. Figure 9 shows the relative variation of the fitting *R*_*g*_, *C*_*g*_ and *C*_*gb*_ values with and without magnetic field, defined as [*M*(*H*) − *M*(0)]/*M*(0)×100%, here *M* denotes as *R*_*g*_, *C*_*g*_ and *C*_*gb*_. We note that an exact single *R*_*g*_ value cannot be unambiguously determined below ~250 K in the fitting process as plotted in Fig. 9(b). It is clear from Fig. 9(a) that the variation of *C*_*g*_ reflecting the intrinsic coupling between spin and dipolar order can reach ~2% at low temperature while decreases dramatically with temperature and becomes faint above about 350 K, indicating the intrinsic ME coupling occurred in BTFCO polycrystals. However, note that the variation of *C*_*gb*_ is more obvious than that of *C*_*g*_, arriving at ~3% even in the low temperature region in Fig. 9(c), and then goes upward with increasing temperature. This MD coupling can be regarded as the interface driven MD effect, such as diodes, grain boundaries *etc.*, attributed to the interactions between the accumulated interfacial charges and magnetic field^27^,^30^. Consequently, these results can clearly shed light on the apparent MD coupling occurred at lower frequency and high temperature regions as shown in Fig. 5(a,c). However, the *R*_*g*_ is also found to increase with the applied magnetic field as given in Fig. 9(b), which might provide some supports to the observed MD coupling arising from the magnetoresistive character from the magnetoimpedance spectroscopy^29^,^30^. In addition, when the frequency of the hole transfer between Cr^+3^ and Cr^6+^ ions and/or the hopping of the electron between Fe^+3^ and Fe^2+^ ions is consistent with the measurement frequency, the concentration of the associated defect charges in the grain interiors would vary significantly, which would bring about the accumulation and/or depletion of interfacial charges in the grain boundaries. This is the main possible mechanism that the interface driven MD effect, *i.e.* the variation of *C*_*gb*_, is more obvious. These data also give a hint that the MD effect coincides with the onset of the relaxation in Fig. 5(b) and remarkable variation of dielectric loss with magnetic field occurs close to the loss peaks in Fig. 5(c,d).

## Conclusion

To summarize, two different dielectric relaxation processes were observed in Aurivillius Cr-doped Bi_5_Ti_3_Fe_0.5_O_15_ polycrystals, which were systemically analyzed by the temperature-dependent dielectric, module, impedance spectroscopy and equivalent circuit model. The two relaxation processes were unambiguously determined to be associated with the localized carrier hopping of electrons between Fe^3+^ and Fe^2+^, and holes between Cr^3+^ and Cr^6+^, respectively. Intrinsic and extrinsic permittivity and MD behaviors were extracted, and the variation of intrinsic permittivity confirmed the ME coupling between spin and lattice occurred in BTFCO polycrystals.

### Experiments

Aurivillius BTFO MF polycrystals with different Cr-doping contents were prepared employing the conventional solid state reaction. The effects of Cr-doping concentrations on the crystal- and micro-structural, magnetic, dielectric and ferroelectric properties of the BTFO polycrystals were investigated in detail in Ref. 30 and 31. Single-phase Cr-doped BTFO polycrystals were determined by a Bruker D8 X-ray diffractometer in the precision limit. BTFCO polycrystals with nominal half substitution of Fe ions by Cr ions (denoted as BTFCO) is selected as a typical case. High-resolution transmission electron microscopy (HRTEM, JEM-2100F, Japan) equipped with the energy dispersive X-ray (EDX) was employed to further analyze the crystalline structure and phase impurity of the BTFCO samples. Signals of valence states of Cr and Fe ions for the BTFCO polycrystals were measured by X-ray photoelectron spectroscopy (XPS, AXIS Ultra DLD, Japan). An Agilent E4980A impedance analyzer was mounted on a physical property measurement system (PPMS-9 Quantum Design) to record the dielectric data at various temperature and magnetic fields.

## Additional Information

**How to cite this article**: Bai, W. *et al.* Dielectric behaviors of Aurivillius Bi_5_Ti_3_Fe_0.5_Cr_0.5_O_15_ multiferroic polycrystals: Determining the intrinsic magnetoelectric responses by impedance spectroscopy. *Sci. Rep.* **5**, 17846; doi: 10.1038/srep17846 (2015).
